# Atypical Presentation of Aortic Dissection in a Young Female and the Utility of Point-of-Care Ultrasound in Identifying Aortic Dissection in the Emergency Department

**DOI:** 10.7759/cureus.27236

**Published:** 2022-07-25

**Authors:** Nidhi Kaeley, Anand Gangdev, Santosh S Galagali, Ankita Kabi, Krishna Shukla

**Affiliations:** 1 Emergency Medicine, All India Institute of Medical Sciences, Rishikesh, Rishikesh, IND; 2 Anesthesiology, All India Institute of Medical Sciences, Gorakhpur, Gorakhpur, IND

**Keywords:** point-of-care ultrasound (pocus), cardiac echo, ct aortogram, atypical presentation of aortic dissection, type a aortic dissection

## Abstract

In the absence of prompt diagnosis and treatment, aortic dissection is an extremely dangerous and often fatal medical condition, of which acute coronary syndrome, stroke, limb ischemia, pulmonary embolism, and acute mesenteric ischemia are all possible manifestations. Neurological manifestations of aortic dissection are often missed at presentation. We report a case of a 23-year-old female without any prior characteristics of connective tissue disorder presenting to the emergency department with headache and right upper limb weakness and the utility of bedside point-of-care ultrasound (POCUS) for diagnosing aortic dissection.

## Introduction

Aortic dissection is an unusual and fatal condition that must be identified and managed promptly for patient survival. The incidence of thoracic aortic dissection is 3-4 in every 100,000 people per year, and if not detected and treated promptly, it can have high mortality [1]. Aortic dissection causes death in as many as 20% of patients who never make it to the hospital. Untreated dissections have a mortality rate of 25% after six hours and 50% after 24 hours; if left untreated, two-thirds of patients will die within a week [2].

Diagnosing an acute aortic dissection can be challenging for a variety of reasons, as it resembles other more prevalent illnesses, including acute coronary syndrome, pulmonary embolism, heart failure, acute mesenteric ischemia, acute limb ischemia, and stroke. In cases of aortic dissection, thrombolysis treatment might be catastrophic, making it critical to distinguish between aortic dissection and acute coronary syndrome.

The most prevalent symptom of aortic dissection is acute chest discomfort that spreads to the back. Pain may be present alone or accompanied by symptoms such as syncope, dyspnea, palpitations, abdominal pain, or neurological deficit. An aortic dissection that causes no discomfort is extremely unusual. In a recent study, women who presented with atypical symptoms were shown to be more likely to have delays in diagnosis [3,4].

A previous study by the International Registry of Aortic Dissection (IRAD) found that 66% of patients with acute aortic dissection were men (mean age: 63.1 years), with women presenting at a significantly older age (mean age: 65.2 years). This is consistent with the findings of other studies. Women between the ages of 30 and 45 with aortic dissection are understudied [5].

This case report highlights the possibility of aortic dissection in younger individuals presenting with atypical symptoms and the utility of bedside ultrasonography in the early detection of aortic dissection and in preventing severe morbidity and mortality.

## Case presentation

A 23-year-old female patient presented to the emergency department with complaints of sudden-onset headache for one day, which was holocranial and associated with two episodes of vomiting without nausea. The vomitus contained food particles and was bilious and not blood-tinged. The patient had associated complaints of palpitations and diaphoresis. The headache was followed by sudden-onset numbness and a tingling sensation of the right upper limb. Thereafter, the patient developed sudden-onset weakness of the right upper limb for two hours, for which she was rushed to the emergency department. There was no history of seizure, loss of consciousness, dysarthria, dysphagia, or bowel or bladder involvement.

Upon examination, the patient was conscious and oriented. Supine blood pressure in the right arm was 68/50 mm of Hg and in the left arm was 134/60 mm of Hg. The patient’s pulse rate was 96 beats per minute, and her respiratory rate was 18 per minute. The right upper extremity was cool to the touch. There was no pallor, cyanosis, clubbing, pedal edema, or lymphadenopathy. Central nervous system examination revealed weakness of the right upper limb (power: 4/5), as compared to the left upper limb (power: 5/5). The power of the bilateral lower limb was 5/5. The tone was increased (spasticity) in the right upper limb and normal in all other limbs. Upon examination of deep tendon reflexes, hyperreflexia was found in the right upper and lower limbs. Extensor plantar response was seen on the right side. Pain and touch sensations were normal bilaterally. Joint position sensation was normal bilaterally. Romberg’s test was positive, and the patient had an ataxic gait. Radial and brachial artery pulsations were feeble in the right arm. All the other peripheral pulses were normal, as were respiratory and abdomen examinations. A cardiovascular system examination revealed an early diastolic murmur in the third left intercostal space. Thrill and heave were absent.

**Figure 1:**
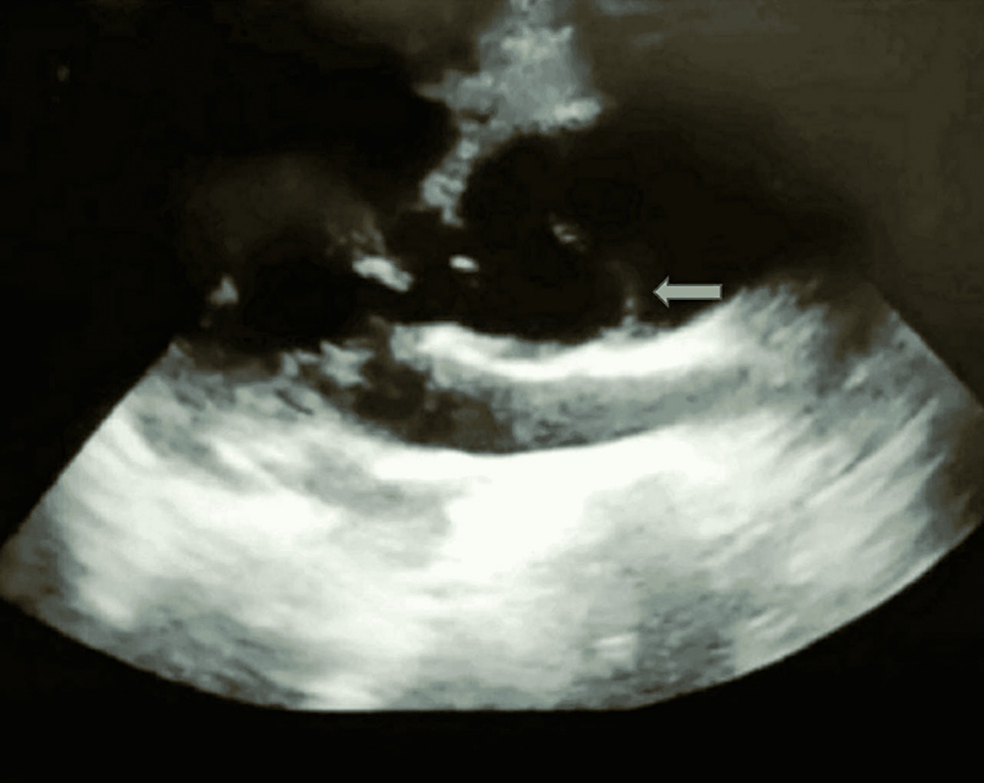
Intimal flap seen on parasternal long-axis view (arrow) (point-of-care ultrasound)

**Figure 2:**
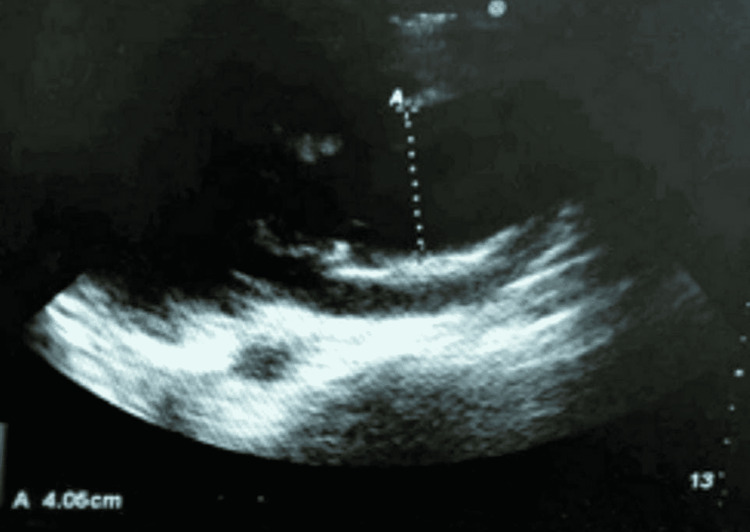
Aortic root diameter of 4.06 cm (point-of-care ultrasound)

**Figure 3:**
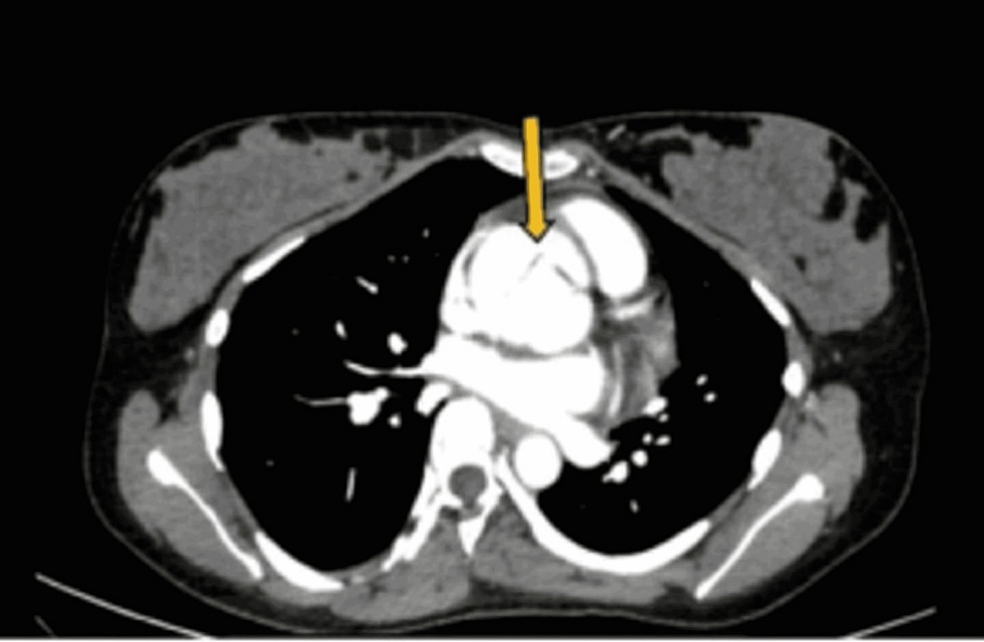
CT aortogram showing Stanford type A aortic dissection (arrow) (axial view)

**Figure 4:**
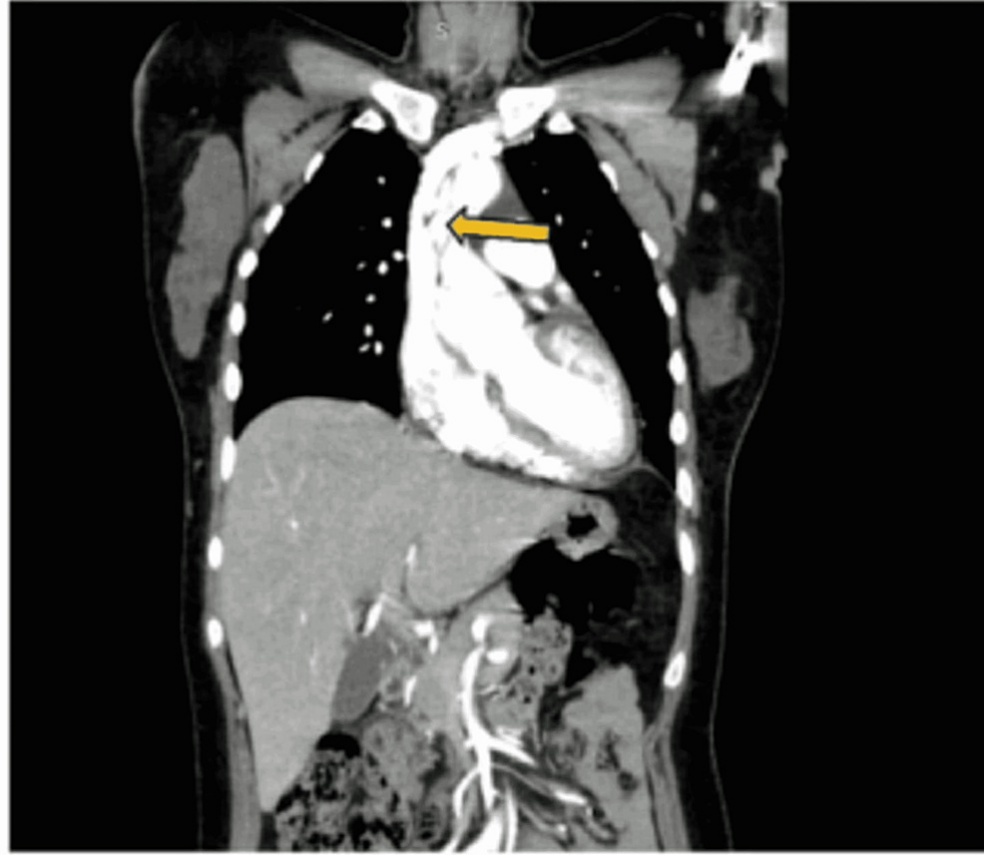
CT aortogram showing Stanford type A aortic dissection (arrow) (coronal view)

An arterial blood gas analysis was done, which was within normal limits (Table 1). An electrocardiogram (ECG) showed sinus tachycardia. Troponin I was negative. A bedside chest X-ray (CXR) was normal. Based on the patient’s acute presentation, point-of-care ultrasonography (POCUS) was performed, which demonstrated aortic root dilatation with a large intimal flap extending from the aortic root (Video 1) (Figures 1, 2), aortic regurgitation, mild pericardial effusion, and a left ventricular ejection fraction of 55%. The patient’s liver and kidney function test results were within normal limits. The complete blood count (CBC) was suggestive of mild anemia, both normocytic and normochromic, with a normal total leukocyte count (Table 1). A computed tomography (CT) aortogram was performed, which confirmed a Stanford type A/DeBakey type 1 aortic dissection and multiple intimal tears in the aortic root, extending up to the ascending aorta and arch of the aorta with the formation of two lumens (Figures 3, 4). The tear extended into the right brachiocephalic trunk and left common carotid artery.

**Table 1:** Laboratory investigations ABG: arterial blood gas; pH: potential of hydrogen; PaCO2: partial pressure of carbon dioxide in arterial blood; CBC: complete blood count; TLC: total leukocyte count; LFT: liver function test; KFT: kidney function test; SGOT: serum glutamate oxaloacetate transaminase; SGPT: serum glutamate pyruvate transaminase

Laboratory parameters	Patient value	Reference range
ABG		
pH	7.40	7.35-7.45
PaO2	88	80-100
PaCO2 (mm of Hg)	33	35-45
Bicarbonate (mmol/L)	20	22-26
Lactate (mmol/L)	2.1	<2
CBC		
Hemoglobin (gm/dL)	10.2	11.5-15
Erythrocyte count (million/mm^3^)	3.60	3.8-4.8
TLC (cells/mm^3^)	4,250	4,000-10,000
Platelet count (thousand/mm^3^)	350	150-450
LFT		
Total bilirubin (mg/dL)	0.42	0.3-1.2
Direct bilirubin (mg/dL)	0.16	<0.2
SGOT (U/L)	24	<35
SGPT (U/L)	15	<35
KFT		
Urea (mg/dL)	29.70	10-43
Creatinine (mg/dL)	0.80	0.5-1

**Video 1:** Intimal flap extending from the aortic root seen on parasternal long-axis view (point-of-care ultrasound)

The patient was managed with labetalol to maintain a pulse rate of <60 beats per minute and systolic blood pressure of <110 mm of Hg. The patient underwent surgical repair via a modified Bentall operation plus hemi-arch replacement. The patient was admitted to the critical care unit following the operation and discharged after 14 days.

## Discussion

Aortic dissection is difficult to detect, particularly in young individuals presenting with atypical symptoms. Among Stanford type A aortic dissection, characteristic tearing-type chest pain is seen in <50% of patients, while approximately 25% of patients have no chest discomfort [4]. Marfanoid habitus, pulse deficit, blood pressure difference, and new-onset aortic regurgitation murmur are all clinical characteristics that may be present. According to the IRAD, nearly half of patients were hypertensive from the start, while the other half had either normal blood pressure or were hypotensive [4]. A variety of symptoms can present depending on how many arterial branches are affected and the relative perfusion to various organs. Coronary insufficiency, intestinal ischemia, renal ischemia, limb ischemia, or weakness may arise and should be assessed in patients with aortic dissection. Loss of consciousness and stroke symptoms are possible scenarios of an aortic dissection affecting brain arteries.

Acute dissection of the ascending aorta requires immediate surgical replacement as a treatment option. This illness has a high mortality risk if left treated. The surgical intervention aims at resecting and replacing the ascending aorta, which eliminates the risk of further dissection in distal parts.

Our patient did not have any signs of marfanoid habits on examination, but it is possible that she was prone to aneurysm growth and dissection due to an underlying connective tissue condition. Typically, younger women with aortic dissection have recognized risk factors, such as Marfan syndrome or past aortic valve disease, and rarely present with neurological deficits [6].

Aortic dissection has been related to neurological symptoms in as much as 14.7% of patients, with as much as 21.8% of those being of Stanford type A (DeBakey type I) dissection, which damages the brain’s major arteries [7]. The most common neurological manifestation was an ischemic stroke and transient ischemic attack [8]. Transient global amnesia, ischemic neuropathy, hypoxic-ischemic encephalopathy, spinal cord ischemia and syndromes, and seizures were among other neurological manifestations [7,8]. Cerebral involvement in the case of aortic dissection can be due to dissection of aortic arch arteries, hypoperfusion due to global hypotension, and/or nerve compression by an expanding lumen. Thrombolysis caused fatal hemorrhagic consequences in three of four patients who got thrombolysis for acute ischemic stroke induced by aortic dissection, according to a review of the literature [7]. Furthermore, few cases of suspected myocardial infarction after thrombolysis have been reported, in which the dissection extended into the pericardium, resulting in cardiac tamponade and mortality [9].

A variety of radiological tests are utilized to aid in the diagnosis of aortic dissection, including CXR, CT with contrast, transesophageal echocardiography (TEE), and magnetic resonance angiogram (MRA). In CXR, 10%-20% of aortic dissections show a widened mediastinum [10]. A transesophageal echocardiogram and a computed tomography (CT) scan with contrast material are the gold standard tests for aortic dissection. Although CT, TEE, and MRI are highly accurate, they are expensive and not commonly available, and require the shifting of critically ill patients from their resuscitation zone.

POCUS provides bedside information about unstable diseases and aids in resuscitation. Several POCUS signs of Stanford type A aortic dissection are highly sensitive: dilated aortic root at end-diastole (>3.5 cm) with a sensitivity and specificity of 77%-91% and 72%-95%, respectively; intimal flap with a sensitivity and specificity of 67%-80% and 98%-100%, respectively; intramural thrombus; pericardial effusions; aortic regurgitation; and color flow in Doppler flowing in true and false lumens [11,12]. A dilated aortic root (Figure 1) and intimal flap (Figure 2) were proved in our case. Using POCUS, emergency doctors were able to correctly identify 88% of Stanford type A aortic dissections, as demonstrated by Nazerian et al. [13]. POCUS can significantly reduce the time it takes to diagnose an aortic dissection (>145 minutes) if performed early, as asserted by Pare et al. [14].

## Conclusions

Aortic dissection is a “great masquerader,” as it can be often mistaken for other cardiac, renal, abdominal, muscular, or neurological diseases. If left untreated, the disease has a high mortality rate. Early diagnosis and management provide survival benefits to patients. The case presented here highlights the likelihood of aortic dissection in young individuals presenting with neurological symptoms without any features suggestive of connective tissue disorder. Bedside ultrasonography can aid in the prompt and early diagnosis of aortic dissection, thereby preventing significant mortality and morbidity.
